# Mean curvature and texture constrained composite weighted random walk algorithm for optic disc segmentation towards glaucoma screening

**DOI:** 10.1049/htl.2017.0043

**Published:** 2018-01-05

**Authors:** Rashmi Panda, N.B. Puhan, Ganapati Panda

**Affiliations:** School of Electrical Sciences, Indian Institute of Technology Bhubaneswar, Bhubaneswar, Odisha 752050, India

**Keywords:** image texture, medical image processing, image segmentation, tumours, cancer, blood vessels, neurophysiology, biomedical optical imaging, mean curvature, texture constrained composite weighted random walk algorithm, optic disc segmentation, cup-disc ratio-based glaucoma screening, fundus imaging, OD boundary, blood vessel occlusion, random walk algorithm, Gabor texture energy features, composite weight function, edge weights, deformable model-based OD segmentation techniques, curve initialisation, local energy minima problem, DRIVE database images, DIARETDB1 database images, DRISHTI-GS database images, MESSIDOR database images, mean absolute distance, overlapping ratio, dice coefficient, quantitative performance

## Abstract

Accurate optic disc (OD) segmentation is an important step in obtaining cup-to-disc ratio-based glaucoma screening using fundus imaging. It is a challenging task because of the subtle OD boundary, blood vessel occlusion and intensity inhomogeneity. In this Letter, the authors propose an improved version of the random walk algorithm for OD segmentation to tackle such challenges. The algorithm incorporates the mean curvature and Gabor texture energy features to define the new composite weight function to compute the edge weights. Unlike the deformable model-based OD segmentation techniques, the proposed algorithm remains unaffected by curve initialisation and local energy minima problem. The effectiveness of the proposed method is verified with DRIVE, DIARETDB1, DRISHTI-GS and MESSIDOR database images using the performance measures such as mean absolute distance, overlapping ratio, dice coefficient, sensitivity, specificity and precision. The obtained OD segmentation results and quantitative performance measures show robustness and superiority of the proposed algorithm in handling the complex challenges in OD segmentation.

## 1 Introduction

Glaucoma is a degenerative and irreversible optic neuropathy, which ranks as the second most disabling and vision impairing disease worldwide [1]. Glaucoma is a silent thief of sight as it is asymptomatic in preliminary stages; hence early diagnosis and treatment is the only way to prevent further retinal damage. Tests such as tonometry, gonioscopy, perimetry are commonly practiced to detect glaucoma. However, these tests are generally time consuming and prone to human errors; therefore computer-aided diagnosis is suitable for large-scale glaucoma screening [2].

There are two approaches for glaucoma detection in the literature such as with segmentation [3–6] and without segmentation [7–15]. In [7–15], the methods use whole image-based features that include higher-order spectral features [7–10], fractal features [11, 12], wavelet-based features [13] and texture features [14] followed by various classification strategies to accurately detect glaucomatic cases. Since this approach does not require explicit segmentation, it is computationally inexpensive. However, the other approach employing segmentation-based reliable features such as cup-to-disc height ratio (CDR), rim area, optic disc (OD) size is also found to be useful for glaucoma screening. The proposed method only concentrates on the OD segmentation procedure which can be used to determine the OD height. The OD height is a prerequisite in computing the CDR-based glaucoma risk index evaluation [16]. To further enhance glaucoma detection accuracy, both approaches can be combined together to design better automated glaucoma detection systems. Moreover, OD segmentation is not only limited to glaucoma detection but also considered to be a fundamental step in diabetic retinopathy detection and localisation of other retinal structures such as fovea and macula [17, 18]. Subtle OD boundary, blood vessel occlusion and intensity inhomogeneity make OD segmentation a challenging task.

**Fig. 1:**
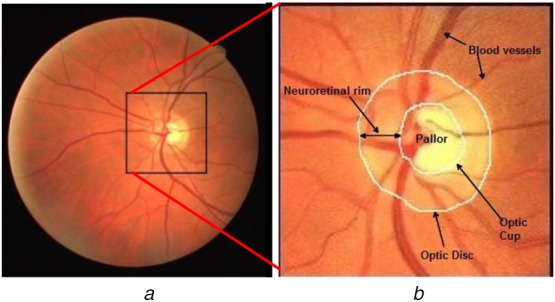
Major retinal structures *a* Colour fundus image (DRIVE database) *b* Enlarged OD region with major retinal structures labelled

OD appears as a bright and relatively circular region, which is partially occluded by the blood vessels as shown in Fig. 1. The approaches adopted for OD segmentation as reported in the literature can be broadly categorised as (i) shape-based template matching, and (ii) deformable model-based methods. In template matching-based methods, the OD is modelled as a circular or elliptical object based on its shape [19–24]. The OD contour is estimated by circular template matching on edge maps using the Hausdorff distance measure [19]. Aquino *et al.* [20] computed the Hough transform-based circular approximation of the OD using a binary mask of the boundary candidates. Another Hough transform-based OD segmentation approach is also reported in [21]. A circular transformation using evenly-oriented radial line segments of specific length is designed in [22] to capture the circular shape of the OD and the image variation across the OD boundary. The ellipse fitting algorithm with intensity information is utilised to detect the OD contour in [23]. A convex hull in the vicinity of best OD candidate followed by an ellipse fitting approach is suggested in [24]. The shape-based template matching methods have reported several failure cases when an OD shape irregularity is caused by different retinal pathologies.

A number of methods have been proposed to address the shape irregularity of OD based on various deformable models [3–6, 25–28]. Xu *et al.* [3] detected the OD contour by improving the original snake algorithm in two aspects: knowledge-based clustering and smoothing. Joshi *et al.* [4] applied active contour model with energy function that includes multi-dimensional features such as intensity, colour and texture. An anchored active contour which was initialised by Hough circle fitted to the edges of the binarised distance map is applied to the OD boundary extraction in [5]. Mittapalli and Kande [6] proposed an active contour model for OD segmentation which incorporates the image information from multiple image channels. An automatic OD boundary detection technique based on morphology and active contour model is proposed in [25]. Wong *et al.* [26] proposed a variational level-set model followed by ellipse fitting operation to obtain the smooth OD boundary. A local deformable model with variable edge-strength dependent stiffness for OD segmentation is used in [27]. Dai *et al.* [28] proposed a PCA-based shape energy which constraints the curve evolution in OD segmentation.

In deformable model-based OD segmentation, the boundary localisation is sensitive to curve initialisation. The curve evolution stops at a local energy minimum if proper initialisation is not undertaken. Various types of local information are used in the level set energy functional to address this problem. The main limitation of the level set methods is that they often require specification of several parameters and it is tedious to tune them, especially when the desired contour does not correspond to a local energy minimum. The above challenges provide space to explore and apply graph-based random walk (RW) segmentation paradigm [29]. The RW provides accurate segmentation output even at weak boundaries in the presence of intensity irregularity. In addition, it avoids the trapping at local minima in deformable models and small cut problem in graph cuts. RW-based segmentation was applied successfully in biomedical images such as left ventricle segmentation in cardiac magnetic resonance images [30] and tumour segmentation in brain and liver images [31].

In this Letter, we propose a new curvature and texture constrained RW (CTCRW) algorithm for OD segmentation in fundus images. Here, we propose a graph-based RW algorithm with a modified weight function which is new for OD segmentation. A new composite RW weight function is defined by incorporating mean curvature, Gabor texture energy features with multiple orientations and intensity features. The distinctive curvature and texture information of OD enable the segmentation process to tackle the misleading interference such as noise, weak boundaries in the OD segmentation procedure.

The remaining of this letter is organised as follows: Section 2 describes the details of the proposed CTCRW-based segmentation method. The results are provided in Section 3. In Section 4, the effectiveness of the proposed CTCRW algorithm is tested and validated by comparing the accurate OD segmentation performance on a wide variety of fundus images taken from DRIVE, DIARETDB1, DRISHTI-GS and MESSIDOR databases. Finally, Section 5 presents the concluding remarks.

## 2 Methods

**Fig. 2:**
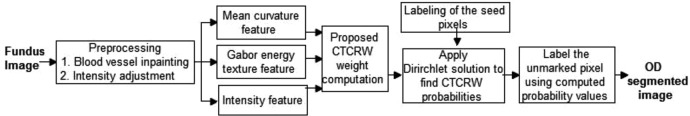
Schematic representation of the proposed OD segmentation algorithm

This section describes the proposed CTCRW algorithm for accurate OD segmentation. The schematic representation of the proposed OD segmentation algorithm is illustrated in Fig. 2. In the first step, blood vessel inpainting and intensity adjustment is performed. The mean curvature feature, Gabor energy texture feature and intensity features are then extracted and used to compute the weights for the proposed CTCRW algorithm. After selecting the background and foreground seed pixels, the solution to combinatorial Dirichlet problem minimisation is computed to get the probability of unmarked pixels belonging to the seed pixels. Finally, the segmentation decision is obtained by retaining the maximum probability value at each pixel.

### 2.1 Pre-processing

The OD segmentation process starts with the grey-scale image I created by a weighted combination of red ($I_{\rm r}$) and green ($I_{\rm g}$) channels of the RGB fundus image to enhance the contrast across the OD boundary [22]

(1) $$I = \alpha I_{\rm g} + \left({1 - \alpha } \right)I_{\rm r}\comma \; \quad {\rm where}\quad \alpha = 0.6\eqno\lpar 1\rpar $$

**Fig. 3:**
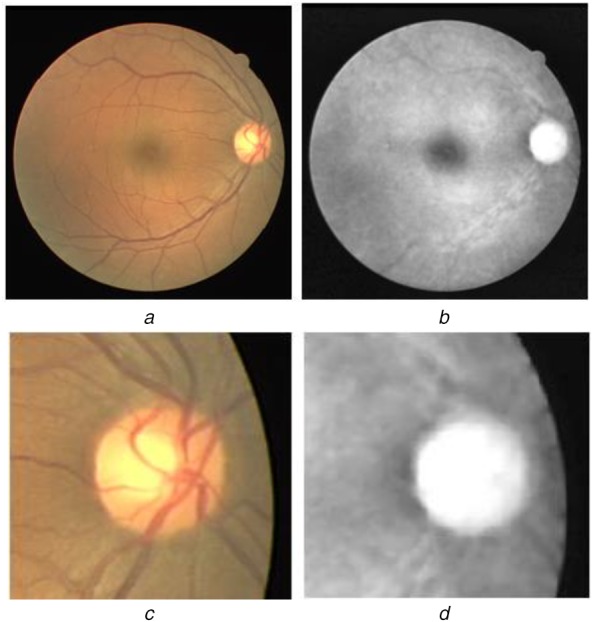
Preprocessing of fundus image *a* Colour fundus image *b* Fundus image after preprocessing *c*, *d* Enlarged OD region of (a) and (b), respectively

The OD segmentation performance degrades due to blood vessel obstruction in and around the OD region. Hence, the blood vessels are extracted using the binary Hausdorff symmetry measure based seeded region growing [32] and then inpainted. This is followed by the intensity adjustment using median filtering at each pixel around the OD centre that is detected based on the symmetry property of retinal blood vessels [33]. A sample pre-processed image is shown in Fig. 3.

### 2.2 RW for segmentation

In RW-based segmentation, an image is treated as a graph with fixed number of vertices and edges. A real-valued weight is assigned to each edge in the graph which corresponds to the likelihood that a random walker will cross that edge [29].

A graph $G = \left({V\comma \; E} \right)$ constitutes of vertices $v \in V$ and edges $e \in E$, where $E \subseteq V \times V$. An edge connecting two vertices $v_i$ and $v_j$ is represented by $e_{ij}$. In a weighted graph, each edge $e_{ij}$ is assigned to a weight $w_{ij}$. The degree $d_i$ of the vertex $v_i$ is the summation of all the incident edge weights

(2) $$d_i = \sum\limits_j {w_{ij}} .\eqno\lpar 2\rpar $$

In classical RW [29], the edge weights are represented by a Gaussian function that maps a change in image intensities to the edge weights

(3) $$w_{ij} = \exp \left({ - \beta {\left({I_i - I_j} \right)}^2} \right)\eqno\lpar 3\rpar $$

where $I_i$ and $I_j$ represent the image intensities at pixels *i* and *j*, respectively, and $\beta $ represents the free parameter of RW algorithm.

The RW algorithm is initialised by assigning *K* seeds indicating regions belonging to *K* objects in the image. The RW algorithm labels each unmarked pixel by computing the probabilities of its first arrival at one among the *K* seed points. A solution using the minimisation of the combinatorial Dirichlet problem with boundary conditions is established to optimally compute the probability values.

### 2.3 Proposed CTCRW algorithm

In this subsection, we present the CTCRW algorithm in details. The CTCRW algorithm uses the mean curvature and Gabor texture energy features as constraints in the proposed RW weight computation. In classical RW, $w_{ij}$ is a function of pixel intensities in (3). However, irrespective of the pathological variations in OD shape, the curvature value is more at the OD boundaries and the texture of OD region is different than the background. Therefore, in addition to pixel intensities, the mean curvature and texture features could achieve improved performance as compared to classical RW. Furthermore, the curvature and texture information in CTCRW is dynamically derived from the image itself such that the requirement of any prior shape training is avoided. The CTCRW algorithm constitutes of mainly three steps: (i) seed initialisation, (ii) assignment of the proposed weight values to the edges, and (iii) computation of the probability of each unmarked pixel belonging to a seed point.

**Fig. 4:**
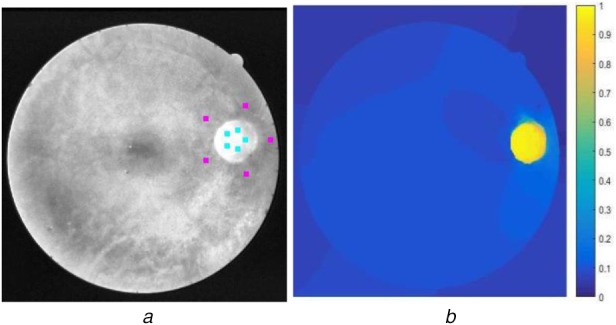
Seed initialization and probability values in CTCRW algorithm *a* Foreground (cyan) and background (magenta) seeds *b* CTCRW probability values

- *Initialisation of seed pixels*: A total of *K* foreground and background seeds are automatically selected on two circles situated around the OD boundary (Fig. 4*a*).
- *Composite edge weight computation*: The edge weights are computed considering the curvature information from the circular shape of OD and texture information from the Gabor energy values.

The mean curvature is an extrinsic measure of curvature that comes from differential geometry and that locally describes the curvature of an embedded surface [34]. The mean curvature *H* of an image *I* is given by

(4) $$H = \displaystyle{{\lpar 1 + I_y^2 \rpar I_{xx} + 2I_xI_yI_{xy} + \lpar 1 + I_x^2 \rpar I_{yy}} \over {2{\lpar 1 + I_x^2 + I_y^2 \rpar }^{3/2}}}.\eqno\lpar 4\rpar $$

Gabor filter is a linear filter used for texture analysis, which analyses the specific frequency content in the image in specific directions in a localised region. A two-dimensional (2D) Gabor filter mask comprises of Gaussian modulated sinusoidal wave used for localised and oriented frequency analysis [35]. A 2D Gabor function for wavelength $\left(\lambda \right)$, orientation $\left(\theta \right)$ and standard deviation $\left(\sigma \right)$ can be expressed as

(5) $$g\lpar x\comma \; y\rpar = \exp ^{ - \lpar {\lpar x - X\rpar }^2 + {\lpar y - Y\rpar }^2\rpar /2\sigma ^2}\sin \left({\displaystyle{{2\pi } \over \lambda }\left({x\cos \theta - y\sin \theta } \right)} \right)\eqno\lpar 5\rpar $$

**Fig. 5:**
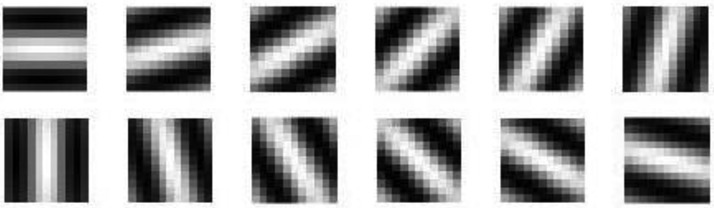
Gabor filters in 12 different orientations

For $g\lpar x\comma \; y\rpar $ computation, the wavelength $\lambda $ and standard deviation $\sigma $ are considered as 1.414 and 0.1, respectively, in 12 equidistant orientations (0, 15, 30, 45,…, 180) in this method (Fig. 5). The Gabor energy is computed by convolving $g\lpar i\comma \; j\rpar $ with the image *I*(*x*, *y*)

(6) $$T_{\theta _i} = I\lpar x\comma \; y\rpar \times g_{\theta _a}\lpar i\comma \; j\rpar \comma \; \quad \theta _a = \lsqb 0^\circ {\rm \comma \; 15}^\circ {\rm \comma \; \ldots \comma \; 180}^\circ \rsqb \eqno\lpar 6\rpar $$

After computing the mean curvature and Gabor texture energy values at different angles, the composite weight of the edge connecting two nodes is found using the following equation:

(7) $$\eqalign{w_{ij} & =c_1\exp \lpar - \beta _1\lpar I_i - I_j\rpar ^2\rpar + c_2\exp \lpar - \beta _2\lpar H_i - H_j\rpar ^2\rpar \cr & \quad + c_3\exp \lpar - \beta _3\sum {{\lpar T_{\theta _ai} - T_{\theta _aj}\rpar }^2} \rpar } \eqno\lpar 7\rpar $$

**Fig. 6:**
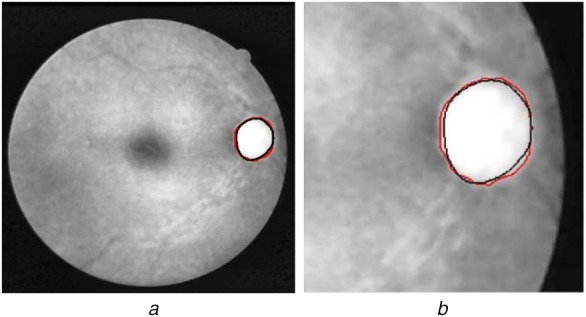
Illustration of OD segmentation *a* OD segmentation result *b* Enlarged OD region with marked contours (red: proposed, black: groundtruth)

- *Labelling of unmarked pixels*: The probability of each unmarked pixel belonging to a seed point is computed by solving the combinatorial Dirichlet problem. The final segmentation decision is obtained by retaining the maximum probability value ($x_u^k $) at each vertex $v_u$ (unmarked pixel)
  (8) $${\rm Labe}{\rm l}_{v_u} = \max _k\lpar x_u^k \rpar \comma \; \quad \forall v_u \in V_u\eqno\lpar 8\rpar $$
  The maximum probability at each unmarked pixel is shown in Fig. 4*b* and the contour of the segmented region is shown in Fig. 6.

## 3 Results

The validation of the proposed CTCRW algorithm is carried out on DRIVE [36], DIARETDB1 [37], DRISHTI-GS [38] and MESSIDOR [39] databases having colour and pathological artefacts variability. The DRIVE database contains 40 colour fundus images including 7 pathological images. The images are captured using 8 bits per colour plane at $564 \times 584$ pixels and 45° FOV (field of view). The DIARETDB1 dataset consists of 89 colour images with 84 of them containing at least one indication of lesion. The images are captured with a digital fundus camera at 50° FOV and had a size of $1500 \times 1152$ pixels. DRISHTI-GS consists of a total of 101 images out of which 70 images have signs of glaucoma. The images are taken with 30° FOV and of dimensions $2986 \times 1944$ pixels. MESSIDOR database [39] comprises of 1200 retinal images captured using 8 bits per colour plane at different resolutions of ${ 1440} \times { 960}\comma \; \, \, { 2240} \times { 1488}\; {\rm or}\; { 2304} \times { 1536}$ pixels.

For quantitative performance evaluation of the proposed algorithm, the following performance measures are taken into consideration. The mean absolute distance (MAD) between the detected OD boundary $I_D$ and the groundtruth $I_G$ is defined as

(9) $${\rm MAD}\lpar D\comma \; G\rpar = \displaystyle{1 \over 2}\left({\displaystyle{1 \over n}\sum\limits_{i = 1}^n {d\lpar d_i\comma \; G\rpar + } \displaystyle{1 \over m}\sum\limits_{\,j = 1}^m {d\lpar g_j\comma \; D\rpar } } \right)\eqno\lpar 9\rpar $$

where *D* and *G* are represented as the sets of contour points, i.e. $D = \lcub d_1\comma \; d_2\comma \; \ldots \comma \; d_n\rcub $ and $G = \lcub g_1\comma \; g_2\comma \; \ldots \comma \; g_m\rcub $ in $I_D$ and $I_G$, respectively. Furthermore, $d\lpar d_i\comma \; G\rpar $ is the distance of point $d_i$ to its closest point in *G*.

The other parameters such as overlapping ratio (OR), dice coefficient (DC), sensitivity (SN), specificity (SP) and precision (PR) values are derived from true positive (TP), false positive (FP), true negative (TN) and false negative (FN) rates as follows:

(10) $${\rm OR} = \displaystyle{{{\rm area}\lpar G_R \cap D_R\rpar } \over {{\rm area}\lpar G_R \cup D_R\rpar }}\eqno\lpar 10\rpar $$

(11) $${\rm DC} = \displaystyle{{2 \times \lpar G_R \cap D_R\rpar } \over {G_R \cup D_R + G_R \cap D_R}}\eqno\lpar 11\rpar $$

(12) $${\rm SN} = \displaystyle{{{\rm TP}} \over {{\rm TP + FN}}}\eqno\lpar 12\rpar $$

(13) $${\rm SP} = \displaystyle{{{\rm TN}} \over {{\rm TN + FP}}}\eqno\lpar 13\rpar $$

(14) $${\rm PR} = \displaystyle{{{\rm TP}} \over {{\rm TP + FP}}}\eqno\lpar 14\rpar $$

where $G_R$ and $D_R$ correspond to the groundtruth and segmented OD regions, respectively.

The performance of the CTCRW algorithm for OD segmentation is first compared with the classical RW [29] and its recent variations applied on medical images [30, 31]. In order to make an unbiased performance comparison, the preprocessing and seed initialisation process are kept identical for each method. In [30], the distance transform of the fitted circle to the initial contour is used in the weighting function to incorporate the circular shape information of the left ventricle during segmentation. An extra penalty term is utilised in the weighting function of [31] which penalises the dispersion of Gaussian-filtered intensities. The segmentation algorithms in [30, 31] are referred as RWDT (RW with distance transform) and RWP (RW with penalty function) here.

**Fig. 7:**
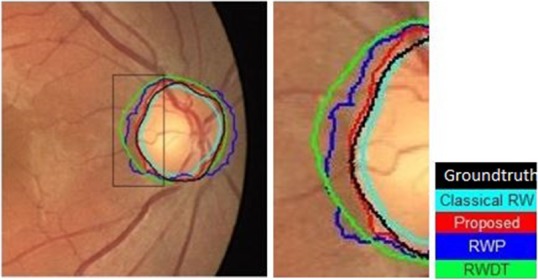
Detected OD boundary

**Fig. 8:**
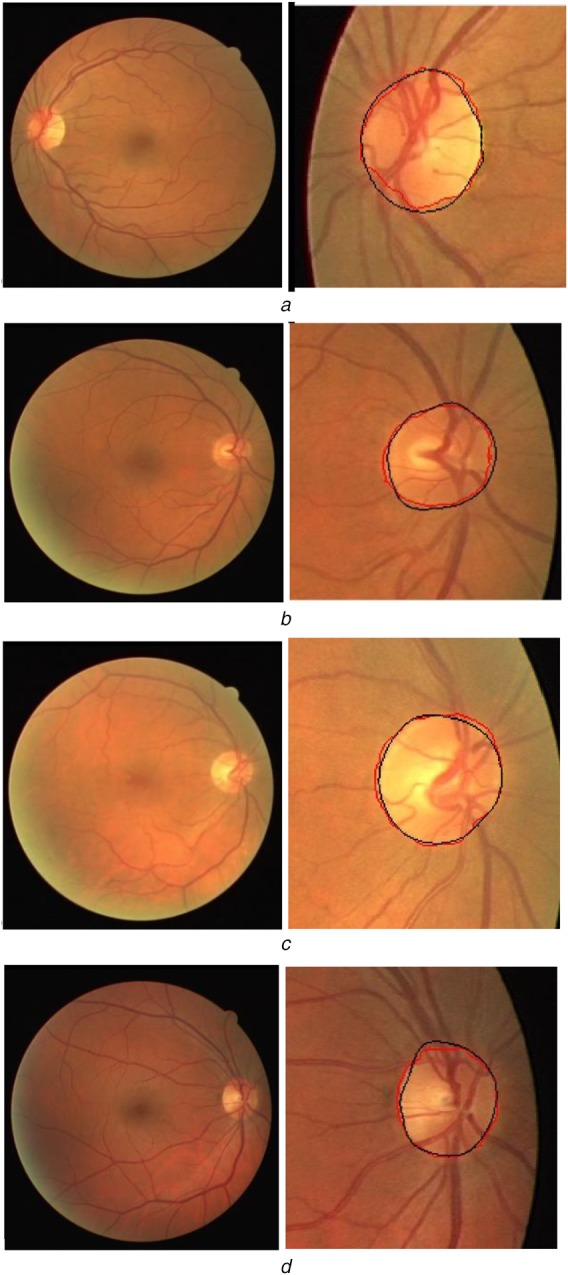
OD segmentation results *a*–*d* First column: colour fundus images and second column: overlapped OD boundaries (black: groundtruth, red: detected contour)

The visual comparison of all these methods is shown in Fig. 7 and the quantitative parameters are given in Table 1. From Fig. 7 and Table 1, it is observed that the proposed algorithm achieves better segmentation performance in terms of MAD (1.46) and OR (0.93) values. This indicates better detection accuracy with an improved match between the groundtruth and proposed segmented outputs. The proposed CTCRW algorithm achieves an improved performance as it incorporates mean curvature and Gabor texture energy features to compute the edge weights. Few more segmentation results are shown in Fig. 8.

**Table 1:** Quantitative parameters

Method	MAD	OR	DC
classical RW [29]	4.51	0.92	0.96
RWDT [30]	5.75	0.75	0.86
RWP [31]	6.33	0.73	0.84
proposed CTCRW	1.46	0.93	0.96

## 4 Discussion

The proposed CTCRW algorithm for OD segmentation incorporates mean curvature, Gabor texture energy features with multiple orientations in the RW weight formulation. Both curvature and texture features of OD are different than the pathological structures like myelinated nerve fibre and peripapillary atrophy. In addition to that, the intensity adjustment around the OD centre in the preprocessing step and careful selection of *K* number of foreground and background seeds add robustness to the proposed method.

The quantitative evaluation parameters computed on complete DRIVE, DRISHTI-GS, DIARETDB1 and MESSIDOR databases are provided in Tables 2–5, respectively. The proposed CTCRW algorithm has MAD values 4.63, 13.03, 7.3 and 2.95 in DRIVE, DRISHTI-GS, DIARETDB1 and MESSIDOR, respectively, which is better than that of classical RW, RWDT and RWP. It signifies that the detected OD contour is nearer to the groundtruth as compared to other methods. It is also observed from Tables 2–5 that the CTCRW algorithm achieves better OR, DC, SN values in all databases. In case of SP of DRIVE and SP, PR of DRISHTI-GS and MESSIDOR, the proposed method has marginally equal performance as compared to RWDT, RWP and classical RW algorithms. The RWDT depends on the circularity criteria; therefore does not perform accurate segmentation where OD is not circular. In RWP, Gaussian filter kernel is used to reduce the responsiveness of the variation of intensities. However, it is unable to detect the correct OD boundary in the presence of peripapillary atrophy around OD. In such challenging scenario, the final OD contours by RWDT and RWP segmentation moves away from the groundtruth.

**Table 2:** OD segmentation performance in DRIVE database

Method	MAD	OR	DC	PR	SP	SN
classical RW [29]	9.89	0.8063	0.8927	0.8964	**0.9985**	0.8871
RWDT [30]	18.47	0.5901	0.7101	0.6136	0.9600	0.9706
RWP [31]	12.06	0.6118	0.7446	0.7207	0.9884	0.8560
proposed CTCRW	**4.63**	**0.8467**	**0.9070**	**0.9257**	0.9983	**0.9167**

**Table 3:** OD segmentation performance in DRISHTI-GS database

Method	MAD	OR	DC	PR	SP	SN
classical RW [29]	15.05	0.8583	0.9222	0.9472	0.9983	0.9041
RWDT [30]	17.40	0.7477	0.8535	**0.9751**	**0.9993**	0.7658
RWP [31]	18.09	0.7370	0.8421	0.9668	0.9989	0.7633
proposed CTCRW	**13.03**	**0.9040**	**0.9496**	0.9441	0.9966	**0.9552**

**Table 4:** OD segmentation performance in DIARETDB1 database

Method	MAD	OR	DC	PR	SP	SN
classical RW [29]	10.08	0.8206	0.8891	0.9029	0.9764	0.9116
RWDT [30]	11.07	0.7344	0.8362	0.9540	0.9910	0.7737
RWP [31]	12.59	0.7367	0.8347	0.9166	0.9765	0.8105
proposed CTCRW	**7.30**	**0.8841**	**0.9385**	**0.9574**	**0.9980**	**0.9203**

**Table 5:** OD segmentation performance in MESSIDOR database

Method	MAD	OR	DC	PR	SP	SN
classical RW [29]	5.88	0.7056	0.8161	0.9091	0.9987	0.7888
RWDT [30]	7.37	0.6511	0.7822	**0.9876**	**0.9999**	0.6580
RWP [31]	10.42	0.6388	0.7575	0.7783	0.9843	0.8434
proposed CTCRW	**2.95**	**0.8588**	**0.9218**	0.9360	0.9994	**0.9168**

Furthermore, Table 6 shows the performance comparisons between the proposed CTCRW approach and state-of-the-art OD segmentation methods. The original results are obtained from the respective papers for this comparison. The proposed CTCRW algorithm achieves OR value of 0.8467 in DRIVE and 0.8841 in DIARETDB1 which is better than the methods in [24, 40–42]. In DRISHTI-GS database, the CTCRW algorithm achieves MAD value of 13.03 which is marginally higher than the MAD value of 11.1 pixels achieved by Joshi *et al.* [4]. In MESSIDOR, the proposed method achieves better performance than Roychowdhury *et al.* [24] in terms of MAD, OR and SN values, whereas Dai *et al.* [28] report slightly higher performance in terms of MAD and OR. The proposed CTCRW algorithm segments the OD more accurately. However, accurate OD centre detection is an additional requirement for successful execution of the proposed segmentation algorithm.

**Table 6:** Performance comparison of the proposed OD segmentation with state-of-the-art methods

Methods	Approach	Database (number of images)	Performance measures		
			MAD	OR	SN
Aquino *et al.* [20]	model-based method	MESSIDOR (1200)	—	0.8600	—
Roychowdhury *et al.* [24]	convex hull at best OD candidate ellipse fitting	DRIVE (40)	5.01	0.8067	0.8780
		DIARETDB0 (130)	4.91	0.7761	0.8660
		DIARETDB1 (89)	4.82	0.8022	0.8815
		STARE (81)	9.13	0.7286	0.8380
		MESSIDOR (1200)	3.93	0.8373	0.9043
Joshi *et al.* [4]	active contour with intensity and texture feature	DRISHTI-GS (101)	11.1	—	—
Mittapalli and Kande [6]	active contour using different image channels	local database (59)	10.11	—	—
Dai *et al.* [28]	shape energy constrained curve evolution	DRIONS (110)	2.42	0.9081	—
		MESSIDOR (1200)	2.25	0.9100	—
Welfer *et al.* [40]	adaptive morphological approach	DRIVE (40)	5.74	0.4147	—
		DIARETDB1 (89)	8.31	0.4365	—
Salazar-Gonzalez *et al.* [41]	Markov random field with compensation factor	DRIVE (40)	3.39	0.8240	0.9819
		DIARETDB1 (89)	6.55	0.7850	0.8750
Diaz-Pernil *et al.* [42]	implementation using parallel architecture	DRIVE (40)	—	0.8330	0.8990
		DIARETDB1 (89)	—	0.8430	0.9180
Muramatsu *et al.* [43]	fuzzy C-mean, active contour and artificial neural network	local test set 1 (98)	—	0.8820	—
		local test set 2 (30)	—	0.8710	—
proposed method	CTCRW	DRIVE (40)	4.63	0.8467	0.9167
		DRISHTI-GS (101)	13.03	0.9040	0.9552
		DIARETDB1 (89)	7.30	0.8841	0.9203
		MESSIDOR (1200)	2.95	0.8588	0.9168

The computation of the probabilities of random walkers first reaching a seed point starting from each pixel is computationally complex. However, it has been established that the minimisation of combinatorial Dirichlet problem makes the RW algorithm simple, convenient and computationally efficient. The proposed CTCRW method is implemented in MATLAB environment on an Intel desktop processor (core i7 CPU, 3.40 GHz). The average computational time per each image for CTCRW is 19.44 s, which is marginally higher than the time of 16.30 s by the classical RW algorithm. In contrast, the processing time of 18.59 and 21.71 s is required for RWDT and RWP-based OD segmentation, respectively. The proposed algorithm has achieved better performance at the cost of few more seconds to compute mean curvature and Gabor texture energy features.

**Fig. 9:**
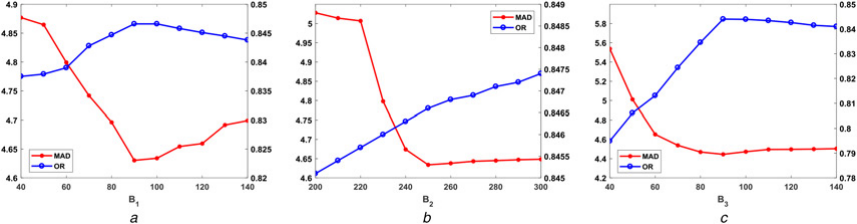
Effect of variation of the parameters on MAD and OR in DRIVE database *a* Varying $\beta _1$ keeping $\beta _2$ and $\beta _3$ fixed *b* Varying $\beta _2$ keeping $\beta _1$ and $\beta _3$ fixed *c* Varying $\beta _3$ keeping $\beta _1$ and $\beta _2$ fixed

The optimal parameter ($\beta _1\comma \; \beta _2\, {\rm and}\, \beta _3$) selection in proposed RW is an important task. The parameters are experimentally chosen to optimise the performance of the segmentation results. The values of the free parameters $\beta _1\comma \; \beta _2\, {\rm and}\, \beta _3$ are chosen as 90, 250 and 90, respectively. To show the parameter SN on result accuracy, each parameter is varied and its effect on the MAD and OR values are shown in Fig. 9 for DRIVE database. The variation of OR and MAD values is compared to a range of each parameter, keeping the other two constants at its optimal value. In Fig. 9, it can be observed that the OR and MAD values attain optimal values at $\beta _1 = 90\comma \; \beta _2 = 250\; {\rm and}\; \beta _3 = 90$. However, once the optimum values of the parameters are decided, it remains fixed for each image in all databases.

## 5 Conclusion

Glaucoma is an irreversible optic neuropathy, which leads to blindness if remains untreated. Early detection and diagnosis of glaucoma can only prevent further vision loss. Monitoring the shape changes in the OD is crucial for indicating the progression of glaucoma. This letter contributes an efficient and fully automated algorithm for accurate OD segmentation. The accuracy of glaucomatous damage estimation depends highly on the exact outlining of the OD contour line. The proposed CTCRW algorithm in this Letter overcomes the curve initialisation and local energy minimum problem of deformable model-based approach. The CTCRW algorithm is shown to segment OD more accurately by incorporating the mean curvature and Gabor texture energy information in the composite edge weight function. The efficacy of the CTCRW algorithm is reflected in terms of the quantitative parameters such as MAD, OR, DC, SN, SP and PR in DRIVE, DRISHTI-GS, DIARETDB1 and MESSIDOR databases. In terms of future work, an accurate optic cup segmentation algorithm will be designed to be used for CDR-based glaucoma classification during the large-scale screening of retinal images.

## 6 Funding and declaration of interests

This work was not funded by any organisation. The authors of this Letter do not have any conflict of interest with any other people or organisations that could inappropriately influence their work.
